# Regulation of Neuronal Na,K-ATPase by Extracellular Scaffolding Proteins

**DOI:** 10.3390/ijms19082214

**Published:** 2018-07-29

**Authors:** Thomas Liebmann, Nicolas Fritz, Markus Kruusmägi, Linda Westin, Kristoffer Bernhem, Alexander Bondar, Anita Aperia, Hjalmar Brismar

**Affiliations:** 1 Science for Life Laboratory, Department of Women’s and Children’s Health, Karolinska Institutet, 17121 Solna, Sweden; tom.liebmann@yahoo.com (T.L.); markus.kruusmagi@gmail.com (M.K.); adnil@kth.se (L.W.); Anita.Aperia@ki.se (A.A.); 2 Science for Life Laboratory, Department of Applied Physics, Royal Institute of Technology, 17121 Solna, Sweden; nicolas.fritz@scilifelab.se (N.F.); kristoffer.bernhem@gmail.com (K.B.); 3 Institute of Chemical Biology and Fundamental Medicine, 630090 Novosibirsk, Russia; alex.bondar@mail.ru

**Keywords:** lateral mobility, clustering, cross linking

## Abstract

Neuronal activity leads to an influx of Na^+^ that needs to be rapidly cleared. The sodium-potassium ATPase (Na,K-ATPase) exports three Na^+^ ions and imports two K^+^ ions at the expense of one ATP molecule. Na,K-ATPase turnover accounts for the majority of energy used by the brain. To prevent an energy crisis, the energy expense for Na^+^ clearance must provide an optimal effect. Here we report that in rat primary hippocampal neurons, the clearance of Na^+^ ions is more efficient if Na,K-ATPase is laterally mobile in the membrane than if it is clustered. Using fluorescence recovery after photobleaching and single particle tracking analysis, we show that the ubiquitous α1 and the neuron-specific α3 catalytic subunits as well as the supportive β1 subunit of Na,K-ATPase are highly mobile in the plasma membrane. We show that cross-linking of the β1 subunit with polyclonal antibodies or exposure to Modulator of Na,K-ATPase (MONaKA), a secreted protein which binds to the extracellular domain of the β subunit, clusters the α3 subunit in the membrane and restricts its mobility. We demonstrate that clustering, caused by cross-linking or by exposure to MONaKA, reduces the efficiency in restoring intracellular Na^+^. These results demonstrate that extracellular interactions with Na,K-ATPase regulate the Na^+^ extrusion efficiency with consequences for neuronal energy balance.

## 1. Introduction

The ubiquitous integral plasma membrane protein Na,K-ATPase exports three Na^+^ ions and imports two K^+^ ions for each ATP hydrolyzed [1,2,3]. In neurons, the energetic costs of action potentials mainly arise from the ATP required for Na,K-ATPase to restore the electrochemical gradients. Approximately 50% of the energy consumed by the brain is used for turnover of Na,K-ATPase [4]. Cellular mechanisms maximizing ion export efficiency are, therefore, highly beneficial. In neurons, the combination of receptor diffusion within dendrites and receptor trapping at synapses constitutes a fast and efficient mechanism for regulation of receptor densities at the synapse and thus synaptic function [5]. Despite the fundamental role of Na,K-ATPase for cellular energy homeostasis, maintenance of resting membrane potential and synaptic function, little is known about the dynamic distribution and regulation of neuronal Na,K-ATPase in the membrane.

The minimal functional unit of Na,K-ATPase is an α/β heterodimer [6]. The α subunit is responsible for the catalytic and ion transport properties of Na,K-ATPase. Neurons express two α isoforms, the neuron-specific α3 and the ubiquitous α1 [7]. Due to its low Na^+^ affinity, α3 is mainly active at the high Na^+^ concentrations reached after synaptic activity [8,9,10]. Mutations in the gene encoding the α3 subunit cause diseases associated with dystonia, cognitive deficits and therapy-resistant epilepsy [11,12]. Many of these mutations are associated with alterations in Na^+^ affinity [13]. The β subunit stabilizes Na,K-ATPase in the endoplasmic reticulum and is necessary for its transport to the plasma membrane [14]. Na,K-ATPase can aggregate to form higher order oligomers in the plasma membrane [15,16]. Mounting evidence indicates that the β subunit plays an important role in control of the oligomerization and sub-cellular distribution of Na,K-ATPase.

Here we have used a combination of nanoparticle, intracellular Na^+^ and super-resolution imaging to characterize mobility and function of Na,K-ATPase in dendrites and excitatory synapses. Extracellular antibody cross-linking of either the α3 or the β1 subunit created clusters of Na,K-ATPase with reduced mobility and reduced Na^+^ transport efficiency. Extracellular exposure to MONaKA, an endogenous protein that specifically binds to the β subunit [17], gave a similar response, with trapping of Na,K-ATPase and a reduced Na^+^ clearance efficiency. Taken together, these results point to a more dynamic and interactive role for Na,K-ATPase in neuronal activity than previously described, and demonstrate a novel regulatory function for the β subunit.

## 2. Results

### 2.1. Lateral Mobility of Na,K-ATPase α3 in Hippocampal Neurons 

**Figure 1:**
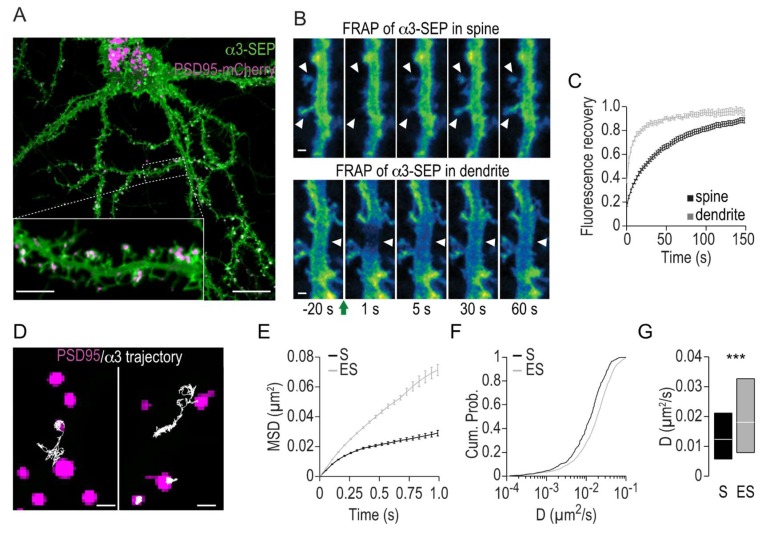
The Na,K-ATPase catalytic subunit α3 is highly mobile in hippocampal neurons. (**A**) Representative confocal picture of a hippocampal neuron (DIV24) transfected with α3 tagged with superecliptic pHluorin (SEP, green) and PSD95-mCherry (magenta). α3-SEP is expressed in dendritic segments and dendritic spines. Scale bars, 20 μm and 5 μm in the inset. (**B**,**C**) Fluorescence recovery after photobleaching (FRAP) analysis of α3-SEP dynamics in dendritic segments and dendritic spines. (**B**) Time lapse images following photobleaching of α3-SEP in dendrites and spines. Images were pseudo-color-coded for SEP intensity. The green arrow indicates photobleaching, and white arrowheads indicate the regions that have undergone photobleaching. Scale bars represent 1 μm. (**C**) Mean ± standard error of the mean (SEM) of the normalized fluorescence intensity after photobleaching over time in dendritic segments (grey) and dendritic spines (black). (**D**–**G**) Lateral diffusion of α3 studied with SPT-QD. (**D**) Representative trajectories (white) of QD-bound α3-SEP in the membrane of hippocampal neurons and over PSD95-mCherry domains (magenta). Left, trajectories exchanging between synaptic and extrasynaptic regions; right, trajectories restricted to either extrasynaptic or synaptic regions. Scale bars represent 1 μm. (**E**) Mean ± SEM mean square displacement (MSD) plots for QD-bound α3-SEP in the extrasynaptic (ES, grey) and synaptic (S, black) regions. Cumulative probabilities (**F**) and median ±25–75% IQR (**G**) of the QD-bound α3 diffusion coefficient D in the extrasynaptic (ES, grey, *n* = 3678 trajectories) and synaptic (S, black, *n* = 610 trajectories) regions. *** *p* < 0.001 with the Mann–Whitney U test.

We first characterized the ensemble mobility of the Na,K-ATPase α3 subunit in the membrane of hippocampal neurons using fluorescence recovery after photobleaching (FRAP). We designed a fusion protein of α3 with superecliptic pHluorin (SEP) inserted in the extracellular loop between transmembrane domains 3 and 4, flanked with flexible linkers to minimize structural interference. Expression of α3-SEP in cultured hippocampal neurons gave a somatodendritic membrane distribution (Figure 1A), in line with what was previously seen with immunolabeling of endogenous α3 [10]. FRAP recordings of α3-SEP were made in dendritic spines and dendritic segments (Figure 1B,C) and mobility parameters were calculated. A large fraction of α3 was mobile, both in dendrites and spines (mobile fraction = 88.7 ± 0.9% in dendrites and 86.3 ± 1.6% in spines). The mobility (characterized by halftime of recovery, t_1/2_) of α3 was lower in dendritic spines than in dendrites (t_1/2_ = 9.1 ± 1.2 s in dendrites and 32.5 ± 4.0 s in spines, *p* < 0.01).

Next, we performed single particle tracking with quantum dots (SPT-QD) in order to gain spatially resolved lateral mobility characteristics of α3 (Figure 1D–G). Hippocampal neurons were co-transfected with α3-SEP and PSD95-mCherry to indicate excitatory synapses. Quantum dots were conjugated to antibodies against SEP. We recorded trajectories of single α3-SEP and used the PSD95-mCherry signal to discriminate the trajectories within synaptic and extrasynaptic regions (Figure 1D). The trajectories were used to quantitatively analyze mobility, expressed as the mean square displacement versus time (MSD(t)) function, and the distribution of diffusion coefficients. In extrasynaptic regions, the MSD(t) function was nearly linear, indicating a largely unrestricted mobility of α3. In contrast, the MSD(t) function was found to be convex in synaptic regions, indicating a restricted mobility of α3 (Figure 1E). Analysis of diffusion coefficients shows that the lateral diffusion is reduced in synaptic regions compared to extrasynaptic regions. (Figure 1F,G).

Taken together, these results of both ensemble and single particle measurements establish that α3 is highly mobile in extrasynaptic membranes of primary hippocampal neurons while the mobility is more restricted at synapses accompanied by a lower diffusion coefficient.

### 2.2. Membrane Distribution and Lateral Mobility of Na,K-ATPase α1 and β1

**Figure 2:**
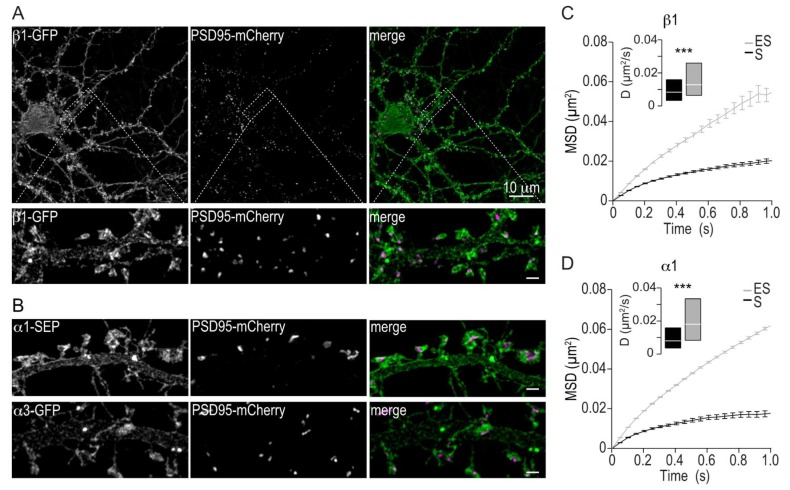
Comparison of α3, α1 and β1 expression and diffusion characteristics. (**A**) Maximum intensity projection of a structured illumination microscopy Z-stack through a DIV24 hippocampal neuron co-expressing β1-GFP (green) and PSD95-mCherry (magenta). Scale bar represents 10 μm. Insets, structured illumination microscopy images of the region in the white rectangle showing dendritic segments and dendritic spines co-expressing β1-GFP together with PSD95-mCherry. Scale bars represent 1 μm. (**B**) Structured Illumination Microscopy images of dendritic segments and dendritic spines co-expressing α1-SEP (top panels, green) or α3-SEP (lower panels, green) together with PSD95-mCherry (magenta). Scale bars represent 1 μm. (**C**,**D**) Mean ± SEM MSD plots for QD-bound β1-GFP (**C**) and α1-SEP (**D**) in the synaptic (S, black lines) and extrasynaptic (ES, grey lines) regions. Insets, median ±25–75% IQR of the QD-bound diffusion coefficients D in the synaptic (black boxes, *n* = 82 trajectories for α1, *n* = 264 for β1) and extrasynaptic (grey boxes, *n* = 360 trajectories for α1, *n* = 871 for β1) regions in cells expressing β1-GFP (**C**) or α1-SEP (**D**). *** *p* < 0.001 with the Mann–Whitney U test.

Hippocampal neurons express the α1, α3 and β1 Na,K-ATPase subunits. We have previously shown that α1 and α3 display similar subcellular expression patterns [10]. To compare in more detail the subcellular distribution of α3 and α1 with that of β1, we used super resolution structured illumination microscopy. An α1-SEP fusion protein was designed similarly to the α3-SEP and a β1-GFP fusion protein was constructed by adding GFP to the extracellular C-terminal of the β1 subunit. We found that β1, similar to α1 and α3, is localized throughout the somatodendritic tree with a distinct expression in dendritic spines (Figure 2A,B). We then measured the mobility of α1 and β1 using SPT-QD. Similar to what was found for α3, both α1 and β1 exhibit a high mobility in the extrasynaptic regions and a more restricted mobility with lower diffusion coefficients in synaptic regions (Figure 2C,D).

Taken together, these results demonstrate that high mobility in extrasynaptic membranes and a more restricted mobility with reduced diffusion coefficients in synapses is a characteristic for all Na,K-ATPase subunits in hippocampal neurons.

### 2.3. Immobilization of β1 Induces Clustering and Immobilization of α3 

**Figure 3:**
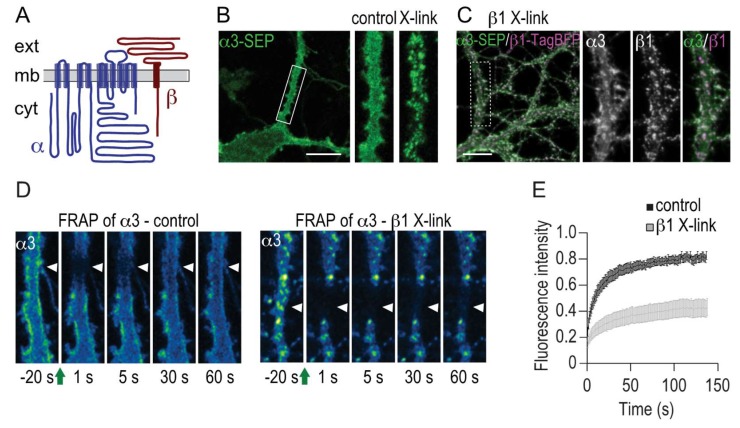
Cross-linking of β1 clusters α3 at the membrane and reduces its membrane dynamics. (**A**) Membrane topology model of the α and β subunit comparing extracellular (ext) and cytoplasmic (cyt) domains. (**B**) Extracellular cross-linking of α3-SEP using goat polyclonal anti-GFP (5 μg/mL, 10 min) and polyclonal donkey anti-goat IgG (10 μg/mL, 10 min) leads to reorganization and clustering of α3 in the membrane. Representative image of a hippocampal neuron at DIV24 expressing α3-SEP. The region in the white rectangle is shown in higher magnification as inset before (control) and after (X-link) cross-linking. Scale bar represents 10 μm. (**C**) Cross linking of β1 clusters α3. Representative image of a hippocampal neuron at DIV24 expressing α3-SEP and β1-TagBFP and subjected to antibody cross-linking against β1-TagBFP with rabbit polyclonal anti-TagRFP (5 μg/mL, 10 min) and polyclonal goat anti-rabbit IgG (10 μg/mL, 10 min). Scale bars represent 5 μm. Single fluorescence channels from the region in the white rectangle are shown in higher magnification as insets. (**D**,**E**) Fluorescence recovery after photobleaching (FRAP) analysis of α3 dynamics in dendritic segments in control conditions (**D**, left) or in dendritic segments subjected to antibody cross linking of β1 (**D**, right). Time lapse images following photobleaching of α3-SEP were pseudo-color-coded for SEP intensity. The green arrows indicate photobleaching, and white arrowheads indicate the regions that have undergone photobleaching. (**E**) Mean ± SEM of the normalized fluorescence intensity over time in dendritic segments in control conditions (black, *n* = 10 cells, 3 cultures) and when β1 is cross-linked (grey line, *n* = 11 cells, 3 cultures).

Interaction of membrane proteins with intra- and extra-cellular factors can lead to aggregation and clustering which influence the lateral mobility of the protein. Notably, the Na,K-ATPase β1 subunit has a large extracellular domain potentially accessible for such extracellular interactions (Figure 3A). To investigate how potential extracellular interactions influence localization, mobility and Na^+^ clearance function of Na,K-ATPase, we performed antibody cross-linking experiments [18]. First, we established that in cells expressing α3-SEP, distinct clusters of α3-SEP are formed by direct cross-linking with polyclonal antibodies against SEP (Figure 3B). To test the secondary effects of crosslinking β1 on α3, a β1-TagBFP fusion protein was designed similarly to β1-GFP and co-expressed together with α3-SEP in hippocampal neurons. Extracellular cross-linking of β1 with polyclonal antibodies for TagBFP resulted in the formation of clusters containing both α3 and β1 with a similar distribution as in the direct α3 crosslinking (Figure 3C). FRAP studies were then performed to assess the size of the mobile pool of α3 when β1 is cross-linked. In each cell, FRAP recordings of both β1-TagBFP and α3-SEP were performed in adjacent dendritic regions (Figure 3D). The mobile fraction of α3 decreased from 79.9 ± 4.5% in control to 33.1 ± 5.8% in β1 cross-linked cells (Figure 3E). In the same cells, the size of the mobile pool of β1 when β1 is cross-linked is 6.2 ± 1.3% demonstrating an efficient cross-linking of β1. The difference in mobile fraction for α3 and β1 indicates a contribution of heterodimers composed of α3-SEP together with endogenous and hence not cross-linked β1. We show here that immobilization of the β1 subunit immobilizes α3. 

### 2.4. Clustering Na,K-ATPase Reduce the Na^+^ Extrusion Efficiency

**Figure 4:**
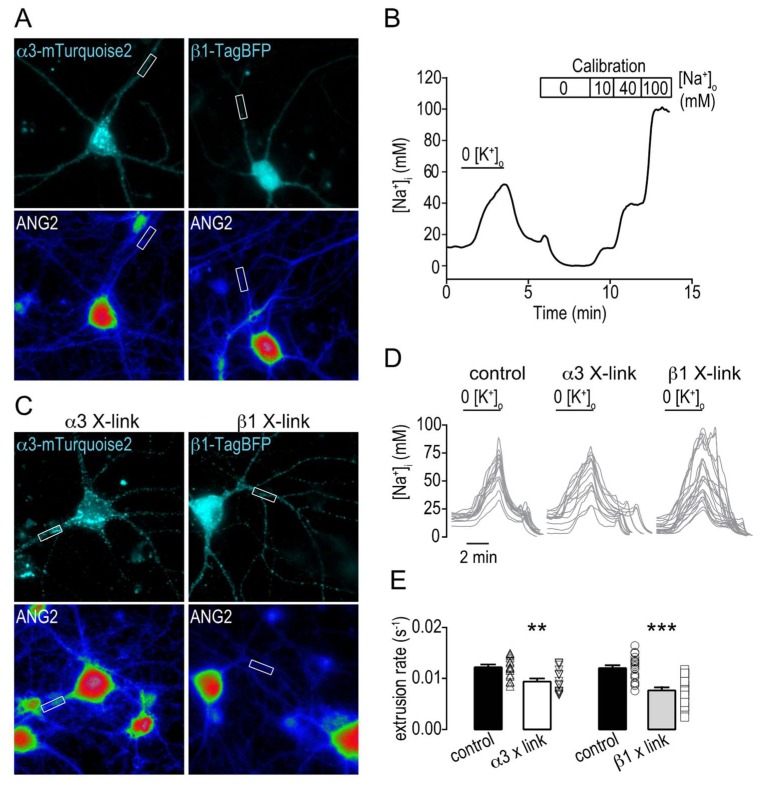
Altered membrane dynamics and clustering of the Na,K-ATPase affects intracellular Na^+^ clearance. (**A**) Representative images of hippocampal neurons in cultures expressing α3-mTurquoise2 or β1-TagBFP and loaded with Asante Natrium Green 2 (ANG-2). The white rectangles indicate typical regions of interests for intracellular recordings of [Na^+^]_i_. (**B**) Typical trace of [Na^+^]_i_ recordings in a single dendrite of a hippocampal neuron challenged with transient perfusion of K^+^-free buffer. Calibration is performed at the end of each experiment. (**C**) Representative images of hippocampal neurons in cultures expressing α3-mTurquoise2 or β1-TagBFP and loaded with Asante Natrium Green 2 (ANG-2), when subjected to antibody cross linking (X-link). The white rectangles indicate typical regions of interests for intracellular recordings of [Na^+^]_i_. (**D**) Example traces of [Na^+^]_i_ recordings in single dendrites of hippocampal neurons challenged with transient perfusion of K^+^-free buffer in control conditions or when subjected to antibody cross linking of α3 (α3 X-link) and β1 (β1 X-link). (**E**) Bar plots of the mean ± SEM Na^+^ extrusion rate in control conditions (black, *n* = 13 dendrites 5 cells for α3, *n* = 19 dendrites 6 cells for β1, 3 cultures) or when subjected to antibody cross linking of α3 (α3 X-link, grey, *n* = 16 dendrites 5 cells, 3 cultures) and β1 (β1 X-link, grey, *n* = 22 dendrites 8 cells, 3 cultures). Individual data values are shown in open symbols for each group. The Na^+^ extrusion rate was defined as the maximum Na^+^ extrusion rate normalized by the maximum [Na^+^]_i_ value during the K^+^-free challenge. Significance was tested with the Mann–Whitney test. ** *p* < 0.01, *** *p* < 0.0001.

We then investigated whether such β1-mediated restriction of α3 mobility would impact the Na,K-ATPase-mediated Na^+^ extrusion efficiency. Here, we used hippocampal neurons expressing β1-TagBFP or α3-mTurquoise2 (constructed similar to α3-SEP) to avoid fluorescence overlap with the Na^+^-sensitive dye Asante Natrium Green-2 (ANG2). Cells were loaded with ANG2 and intracellular Na^+^ was recorded in primary or secondary dendritic segments (Figure 4A). A transient increase of intracellular Na^+^ was induced by perfusing cells with a K^+^-free buffer that inhibits Na,K-ATPase mediated Na^+^ extrusion. Upon subsequent perfusion with normal K^+^-containing buffer, Na,K-ATPase activity resumed. The rate of Na^+^ extrusion was then determined from the calibrated ANG2 signal (Figure 4B and see methods section). Expressed α3-mTurquoise2 or β1-TagBFP was selectively cross-linked to immobilize Na,K-ATPase and the formation of clusters at the membrane was verified (Figure 4C). The Na^+^ extrusion rates were then measured and found to be reduced by 23 ± 9% when α3-mTurquoise2 was cross-linked and by 36 ± 8% when β1-TagBFP was cross-linked (Figure 4D,E).

### 2.5. Clustering of the Neuronal Na,K-ATPase Mediated by MONaKA Binding to the β1 Subunit

**Figure 5:**
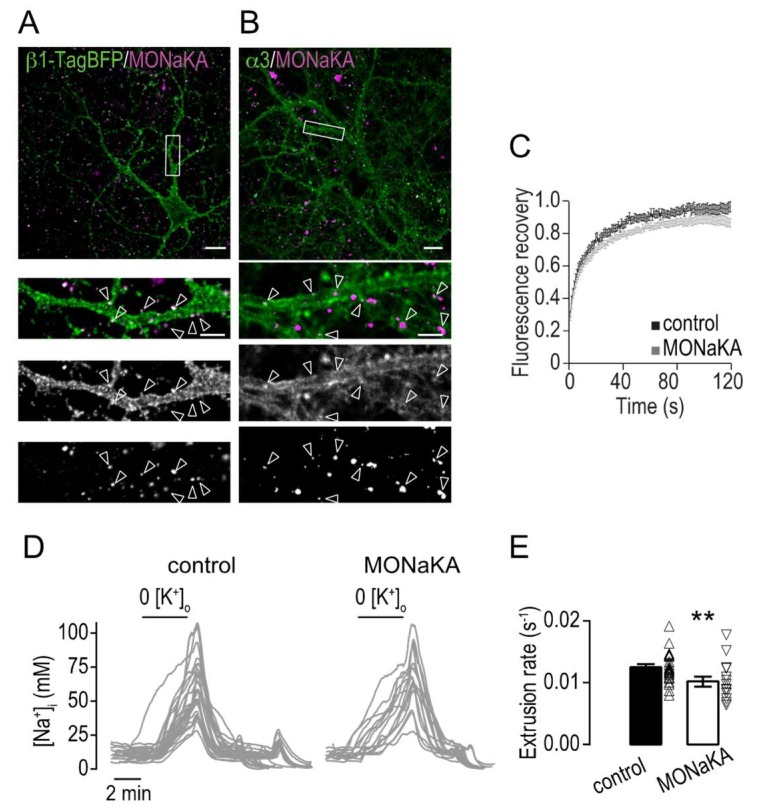
Clustering of Na,K-ATPase by MONaKA affects intracellular Na+ clearance. (**A**) Representative confocal images of hippocampal neurons expressing β1-TagBFP incubated with *myc*-tagged MONaKA (0.1 μM for 24 h). Scale bar represent 20 μm. Insets, higher magnification of the single fluorescence channels from the regions in the white rectangles. Scale bars represent 5 μm. White arrows indicate overlap of α3 and MONaKA clusters. (**B**) Representative confocal images of hippocampal neurons incubated with *myc*-tagged MONaKA (0.1 μM for 24 h) and stained for c-*myc* and endogenous α3. Scale bar represent 20 μm. Insets, higher magnification of the single fluorescence channels from the regions in the white rectangles. Scale bars represent 5 μm. White arrows indicate overlap of α3 and MONaKA clusters. (**C**) Mean ± SEM of the normalized fluorescence intensity after photobleaching over time in dendritic segments of neurons incubated with vehicle (control, black, *n* = 17 cells, 3 cultures) or MONaKA (0.1 μM, 24 h, grey, *n* = 25 cells, 3 cultures). (**D**) Example traces of [Na^+^]_i_ recordings in single dendrites of hippocampal neurons challenged with transient perfusion of K^+^-free buffer in control conditions or when treated with MONaKA (0.1 μM, 24 h). (**E**) Bar plots of the mean ± SEM Na^+^ extrusion rate in control conditions (black, *n* = 29 dendrites, 14 cells, 3 cultures) or when treated with MONaKA (0.1 μM, 24 h, white *n* = 14 dendrites, 6 cells, 3 cultures). Individual data values are shown in open symbols for each group. The Na^+^ extrusion rate was defined as the maximum initial Na^+^ extrusion rate normalized by the maximum [Na^+^]_i_ value during the K^+^-free challenge. Significance was tested with the Mann–Whitney test. ** *p* < 0.01.

The MOdulator of Na,K-ATPase (MONaKA) is a secreted protein expressed in neurons and astrocytes in the brain. Co-immunoprecipitation and pull-down experiments have demonstrated that MONaKA binds to the extracellular domain of β1 [17,19]. To study the effects of MONaKA on the distribution and mobility of Na,K-ATPase we designed a fusion protein with a c-*myc*-tag inserted in the N-terminus of MONaKA. MONaKA was produced, purified and applied (0.1 μM) to neuronal cultures for 24 h. Immunolabeling revealed a formation of MONaKA clusters at neuronal membranes over the whole somatodendritic tree (Figure 5A). The effects of MONaKA treatment on the distribution of α3 was then studied using double labeling for α3 and MONaKA. A redistribution of α3 into larger clusters, often overlapping with or in close proximity to MONaKA, was observed (Figure 5B).

We then performed FRAP experiments on primary hippocampal neurons expressing α3-SEP to test if MONaKA-induced clustering of α3 also affects the mobility. In neurons treated for 24 h with MONaKA, the mobile fraction of α3 was reduced from 89.8 ± 2.5% to 75.8 ± 2.5% (Figure 5C). To test if this reduction in mobility and clustering by MONaKA influence the Na^+^ transport efficiency, as shown in the cross-linking experiments, we determined the Na^+^ extrusion rate in non-transfected hippocampal neurons and found it to be reduced by 18 ± 10% after 24 h of MONaKA treatment (Figure 5D,E).

We show here that MONaKA forms clusters at the membrane of hippocampal neurons leading to clustering and reduced mobility of Na,K-ATPase with a reduced Na^+^ transport efficiency.

## 3. Discussion

We demonstrate here that Na,K-ATPase is highly mobile in neuronal membranes and that impairment of Na,K-ATPase mobility by extracellular trapping of the β1 subunit reduce the efficiency of Na^+^ extrusion. Given the major burden imposed by Na,K-ATPase on cellular bioenergetics, a reduced Na^+^ extrusion efficiency will not only affect Na^+^ homeostasis but also overall neuronal energy balance.

In this study we have used exogenously expressed fluorescent proteins combined with the α3 or β1 subunits of Na,K-ATPase. Care was taken to avoid interference with the structural changes required for the ion transport function of Na,K-ATPase by flanking the fluorescent protein with short flexible arms and inserting it into the extracellular segment between the third and fourth trans-membrane domains of the rat α3 subunit. The similar expression patterns of exogenously transfected and endogenously expressed α3, as well as the similar Na^+^ extrusion rates in transfected and non-transfected neurons provide evidence for a conserved assembly, trafficking and function of exogenous Na,K-ATPase. Cross-linking using polyclonal antibodies has previously been used to investigate clustering and regulation of membrane receptor mobility in neurons [18,20]. To further avoid interference with the function of Na,K-ATPase we here used antibodies targeting the fluorescent proteins rather than the Na,K-ATPase.

In the synapse, we found that Na,K-ATPase has a restricted mobility and reduced diffusion coefficient compared to in the dendrite, well in line with the synaptic clustering observed using super-resolution microscopy [21,22]. This is also consistent with previous reports based on SPT-QD for several neuronal receptors and transporters, including AMPAR [23,24], mGluR5 [25], nAChRα3 and 7 [26] and KCC2 [27]. 

Previous studies have shown that the β subunit is important for the subcellular localization of Na,K-ATPase. It is well established that the β subunit is required for export of the α subunit to the membrane [6,28]. In Madin–Darby canine kidney epithelial cells, the basolateral expression of Na,K-ATPase depends on the β subunit [29,30,31]. Na,K-ATPase can aggregate to form higher order oligomers in the plasma membrane [15,16]. Work from Vagin and Schwartz and colleagues have unraveled an important role for the β subunit in the establishment and consolidation of epithelial cell junctions [32,33,34,35]. The β1 subunit has a glycine zipper motif in the transmembrane domain that enables β1-β1 interaction and oligomerization of the Na,K-ATPase [36]. Such *cis* homo-oligomerization is important for lumen formation in kidney epithelial cells [37]. An interaction between β1 subunits may predispose the formation of Na,K-ATPase oligomers and clusters. 

We demonstrate here that the β1 subunit also plays an important role for regulation of Na,K-ATPase mobility in the membrane. We found that extracellular cross-linking of β1 resulted in formation of clusters containing both β1 and α3. FRAP experiments of fluorescently tagged-α3 in β1 cross-linked neurons confirmed that a large pool of α3 is trapped together with β1 in the membrane. 

One of the major functions of the Na,K-ATPase is to actively maintain intracellular Na^+^ homeostasis by Na^+^ extrusion [38,39]. In neurons, the α3 isoform, which has a lower Na^+^ affinity than the α1 isoform, is the main responsible subunit for Na^+^ transport after neuronal activity [10]. We show here that the efficiency in Na^+^ extrusion is reduced by extracellular constraints to Na,K-ATPase membrane mobility.

The effect of clustering on Na^+^ extrusion efficiency may have serious consequences. A tight control of intracellular Na^+^ concentration is required for precise information processing, synaptic function and behavior. Na,K-ATPase is responsible for the generation of the after-hyperpolarization observed after long trains of action potentials [40,41], and thus contributes to the control of neuronal excitability. Mice carrying loss-of-function mutations in the *ATP1A3* gene encoding for α3 have a reduced threshold for induction of epileptiform activity [42] and, in humans, loss-of-function mutations in the *ATP1A3* gene are associated with a variety of neurological symptoms and cognitive deficits [12]. Recently, a number of pathological factors have been identified that extracellularly interact with Na,K-ATPase. Amylospheroids, 10–15 nm spherical amyloid-beta oligomers derived from Alzheimers disease patients, have been shown to impair the activity of Na,K-ATPase by binding to the fourth extracellular loop of α3 [43]. Similarly, clusters of α-synuclein implicated in Parkinsons disease [44] have been shown to trap α3 resulting in clustering of Na,K-ATPase and impaired Na^+^ extrusion capacity. 

We show here that the non-pathological protein MONaKA can form clusters at the neuronal membrane which trap Na,K-ATPase and reduce the efficiency of Na^+^ extrusion. MONaKA is expressed in the central and peripheral nervous systems and binds to a distinct site of the extracellular domain of the β1 subunit [17]. The molecular mechanisms of MONaKA binding and formation of Na,K-ATPase aggregates remains to be elucidated. It can, however, be speculated that trapping the beta subunit may promote a beta–beta interaction which predisposes formation of larger Na,K-ATPase oligomers. 

Our findings suggest that extracellular interaction and trapping of Na,K-ATPase which lead to a reduced Na^+^ extrusion efficiency may be a mechanism present not only in pathologies but also a possibility for physiological regulation of the efficiency in Na^+^ extrusion by Na,K-ATPase. One can speculate that altered mobility of the Na,K-ATPase may also locally influence intracellular sodium, and functional interaction of the Na,K-ATPase with other signaling pathways, thus introducing a novel mechanism of inter-cellular regulation of excitability in neurons. 

Finally, our study shows that the mobility of Na,K-ATPase in neuronal membranes is important for the efficient use of ATP. Altered efficiency in ion extrusion will not only affect Na^+^ homeostasis but also the spatio-temporal use of energy by Na,K-ATPase, a parameter that directly affects the neuronal energy balance. In pathological situations, a disruption of the energy balance can lead to an energy crisis which serves as a basis for many neurological disorders [45,46].

## 4. Materials and Methods

### 4.1. Cell Culture and Transfection 

Hippocampal neurons were cultured from E18 Sprague Dawley rat embryos as described previously [47]. Briefly, hippocampi were removed and washed in Hanks’ Balanced Salt Solution with 20 mM HEPES (Sigma-Aldrich, St. Louis, MO, USA) incubated in 0.25% Trypsin (10 min, 37 °C), then dissociated by pipetting in Minimum Essential Medium (MEM). Cells were seeded at a density of ≈1.0 × 10^4^/cm^2^ onto glass coverslips coated overnight at 37 °C with 80 μg/mL poly-dl-ornithine (Sigma-Aldrich). Seeding medium contained MEM with 10% horse serum, 2 mM l-glutamine, and 1 mM sodium pyruvate (Sigma-Aldrich). After 3.5 h, the medium was replaced with Neurobasal medium containing 2% B27, 2 mM l-glutamine, 1% penicillin/streptomycin (Sigma-Aldrich). Half the medium volume was changed twice a week and replaced with Neurobasal medium as before but with 0.5 mM l-glutamine. Media components were from Gibco, Invitrogen unless otherwise stated. Neurons were transfected after 16–17 DIV with Lipofectamine 2000 (Invitrogen, Carlsbad, CA, USA) according to the manufacturer’s recommendations. Experiments were performed at 22–25 DIV to allow for synaptic maturity.

### 4.2. Construct Design

Expression plasmids for Na,K-ATPase subunits with terminal tags were generated by polymerase chain reaction (PCR) amplification of each pump cDNA sequence (rat ATP1a3 and ATP1b1) and insertion into EGFP, PAGFP, TagBFP or TagRFP vectors. α and β subunits were cloned into –C1 and –N1 vectors, respectively. EGFP and TagXFP vectors were purchased from Clontech and Evrogen, respectively. Internal α tags on the α subunit were made by first generating insertion sites by site-directed mutagenesis between W307/L308 (ATP1a3) or W317/L318 (ATP1a1). Superecliptic pHluorin (SEP) and mTurquoise2 were subcloned with flanking flexible linkers (GGGGS) into the insertion sites. The construct for fluorescent PSD-95 was designed as previously reported [22]. The sequences of all constructs were confirmed by sequencing analysis. Amplified DNA was purified with the endotoxin-free PureYield Plasmid MidiPrep System (Promega, Madison, WI, USA). 

### 4.3. Protein Purification

An expression plasmid for modulator of Na,K-ATPase (MONaKA) was made by PCR amplification of the rat long form from cDNA and inserted into a bacterial expression vector, pTrcHis2 TOPO TA expression kit (Life Technologies, Carlsbad, CA, USA) which contained a C-terminal c-*myc* tag for immunolabelling and a N-terminal polyhistidine tag for nickel chelating resin purification. Growth and purification was performed by the Protein Science Facility at Karolinska Institutet/Science for Life Laboratory, Stockholm. Cultivation was done with BL21 (DE3) R3 pRARE2 in 1500 mL Terrific Broth (1.5:100 inoculation ratio) at 37 °C and supplemented with 8 g/L glycerol, 50 μg/mL Ampicillin and 34 μg/mL chloramphenicol. Once an optical density of 2 was reached, temperature was reduced to 18 °C and expression was induced with 0.5 mM IPTG overnight. Cells were harvested by 10 min centrifugation at 4430× *g*. Cells were re-suspended in IMAC lysis buffer and complete stock solution (1 tablet Complete EDTA-free (protease inhibitor cocktail, Roche) and 5 μL benzonase nuclease (250 U, Sigma-Aldrich) per 1 mL). Cells were lysed by sonication (4 s/4 s, 3 min, 80% amplitude) and centrifuged for 20 min at 49,000× *g*. Soluble fractions were filtered through 0.45 μm filters and loaded on ÄKTA Xpress, with 1 mL HisTrap HP IMAC column and HiLoad 16/60 Superdex 200 gel filtration column (all from GE Healthcare, Chicago, IL, USA). Product was concentrated with a 30 kD cutoff to 5.3 mg/mL in batch buffer (20 mM HEPES, 300 mM NaCl, 10% glycerol, 2 mM TCEP, pH 7.5). The purity of each MONaKA batch was confirmed by sodium dodecyl sulfate polyacrylamide gel electrophoresis (SDS-PAGE) and mass spectrometry.

### 4.4. Microscopy

Confocal imaging was performed on an inverted Zeiss LSM 780 microscope with a 63× (1.4 NA, oil) objective. SPT-QD and Na^+^ imaging experiments were performed on an inverted Zeiss Axiovert 200 with 63× (1.4 NA, oil) and 40× (1.3 NA, oil) objectives, respectively, an Andor iXon+ 897 EMCCD camera and an X-Cite lamp. SPT and Na^+^ imaging was made with 30% and 10% illumination intensity, respectively, each with additional neutral density filtering. The QD-655-B filter set was used for QDs; the GFP-3035B filter set for GFP/SEP; the BFP-A-Basic-ZHE for BFP; excitation filter 436/25, dichroic beamsplitter FT455, and emission filter 480/40 for mTurquoise2; excitation filter FF01-543/22, dichroic mirror FF562-DI03, and emission filter FF01-593/40 for mCherry; excitation filter FF03-510/20-25, dichroic beamsplitter Di02-R514-25x36, and emission filter FF01-571/72-25 for Asante naTRIUM green 2 (ANG-2). All custom filters were purchased from Semrock (Rochester, NY, USA). 

### 4.5. 3D Structured Illumination Microscopy (3D-SIM)

Transfected cells were fixated in 4% paraformaldehyde for 10 min, washed 3 × 5 min in PBS and mounted in Prolong Gold. 3D-SIM imaging was performed using a Plan-apochromat 63×/1.4 NA oil objective on an ELYRA PS.1 (Carl Zeiss, Oberkochen, Germany) microscope. Structured excitation was achieved using a 488 nm and a 561 nm laser and a grating period of 28 and 34 μm respectively in 3 rotations at 5 phases. Emission was collected through 495–575 nm and 570–650 nm bandpass filters. Z-stacks were acquired to cover the full dendritic tree in the field of view. High resolution images were reconstructed using the automatic settings in the SIM processing module in ZEN software (Carl Zeiss).

### 4.6. Immunocytochemistry and Live Cell Imaging

Immunolabelling was performed as described previously [10], with a few exceptions. Fixation was performed with 4% paraformaldehyde (room temperature, 5 min) followed by 10% Trichloroacetic acid (room temperature, 5 min) for Na,K-ATPase immunocytochemistry, and with 4% paraformaldehyde (room temperature, 10 min) in other cases. When applicable, cells were permeabilized with 0.2% Triton-X (2–5 min, room temperature), followed by blocking in 10% bovine serum albumin (BSA, 10%, room temperature). Primary antibodies were mouse anti-ATP1a3 (MA3-915, Affinity Bioreagents, San Francisco, CA, USA, 1:2000), mouse anti-c-*myc* (clone 9E10, sc40, Santa Cruz, Santa Cruz, CA, USA, 1:1000), rabbit anti-c-*myc* (C3956, Sigma-Aldrich, 1:1000), rabbit anti-TagRFP (R10367, Thermo Fischer Scientific, Waltham, MA, USA, 1:500), rabbit anti-GFP (600-401-215, rockland, 1:500). Primary antibodies were applied in PBS with 5% BSA for 1–2 h at room temperature. Secondary labelling was done with Alexa 488-, Alexa 568-, Alexa 647-, or Atto 800-conjugated goat anti-rabbit or anti-mouse IgG (Life Technologies, 1:200–1:1000, 1–2 h at room temperature). Coverslips were mounted in Prolong-Gold (Thermo Fischer Scientific, Waltham, MA, USA) and allowed to set overnight before imaging. Live cells were imaged in HEPES-buffered saline (HBS: (in mM) 110 NaCl, 137 NaH_2_PO_4_, 84 NaHCO_3_, 10 glucose, 20 HEPES (Sigma-Aldrich), 75 KCl, 147 CaCl_2_, 203 MgCl_2_ (Merck, Kenilworth, NJ, USA) at pH 7.4) at 37 °C. MONaKA (0.1 μM) was applied to neurons in culture medium. Cross-linking was performed by serial labelling with goat anti-GFP antibodies (5 μg/mL for 10 min, Rockland, for α-SEP) or rabbit anti-TagRFP (1:500 for 10 min, Life Technologies, for ^®^1-TagBFP) followed by unconjugated or Alexa405-conjugated donkey anti-goat or goat anti-rabbit secondary antibodies (10 μg/mL for 10 min, Life Technologies) with three washes with HBS after each labelling step. Control experiments were performed in the presence of secondary antibodies only.

### 4.7. Fluorescence Recovery after Photobleaching (FRAP)

Hippocampal neurons at DIV20-25 expressing α3-SEP, or α3-SEP and ^®^1-TagBFP were used. A single plane through a dendritic segment with visible spines was imaged at 1 Hz in HBS at 37 °C using a Zeiss LSM780 confocal microscope and a 40× (1.3 NA, oil) lens. Squared regions of interest (ROI, 4 μm^2^) were defined so as to encompass whole spine heads or the whole width of the dendritic segment. Neurons were imaged pre-bleach to establish a baseline, followed by photobleaching and recording of fluorescence recovery. Photobleaching was achieved with repeated *xy* scans of the ROI at high illumination intensity using the 405 and 488 nm lasers. Recordings were analyzed using ImageJ (https://imagej.net) and custom-written MATLAB scripts (MathWorks, Natick, MA, USA). The average fluorescence intensity in each ROI was calculated and plotted over time. The data were background-subtracted, corrected for acquisition bleaching and normalized to the intensity before photobleaching. Half recovery times (t_1/2_) were derived by fitting the recovery curves with a double-exponential model, the simplest model that fitted the recovery curves. The percentage of recovery was defined as follows: % recovery = (*F*_p_ − *F*_b_)/(*F*_0_ − *F*_b_) × 100, where *F*_p_ is the intensity of fluorescence at the plateau of recovery, *F*_b_ is the intensity of fluorescence immediately after photobleaching and F_0_ is the intensity of fluorescence pre-bleach.

### 4.8. Single Particle Tracking

QD labelling of transfected cells was performed with biotinylated goat anti-GFP antibodies (Rockland, 4–10 ng/mL, 5 min) and Streptavidin-conjugated quantum dots (Q10123MP, Life Technologies, 0.5 nM, 1 min in QD binding buffer [48]). Cells were then washed briefly 10 times with HBS, and immediately imaged up to 30 min after completion of labelling. After acquiring α3-SEP and PSD95-mCherry images, QD images were recorded with a frequency of 20 Hz for 50 s. All labelling, washing and recording steps were performed at 37 °C. Custom MATLAB (MathWorks, Natick, MA, USA) software was used to identify and trace the QDs. Sub-pixel accuracy (≈15 nm resolution) was achieved by fitting a two-dimensional Gaussian function to each identified QD in every image frame. MSD curves were calculated from reconstructed particle trajectories with >15 positions [49]. Synaptic regions were identified as regions positive for PSD95-mCherry after running fluorescent PSD95-mCherry images through a stationary wavelet transform. Initial diffusion coefficients were calculated by fitting a straight line to points 2 through 5 (interval 50–200 ms) of the MSD curve. Cumulative frequency distributions were calculated from the individual diffusion coefficients. For each experiment, at least 5 transfected cells from 2–5 cultures were analysed.

### 4.9. Na^+^ Extrusion Measurements

Na^+^ Recordings were performed on primary hippocampal neuronal cultures on day in vitro 21–25. Cells were loaded with the acetoxymethyl ester derivative forms of the Na^+^ sensitive ANG2 (Asante NaTRIUM Green 2, TEFLabs; 37 °C, 5% CO_2_) at 1 μM for 25 min in the cell culture medium. A washing step in which coverslips were placed on a hot plate maintained at 37 °C for 20 min in HBS was then performed. For imaging, coverslips were mounted on a heated chamber attached with a perfusion system for rapid exchange of solutions. ANG2 fluorescence was excited at 490 nm and collected above 500 nm, using a Zeiss Axioscope Observer D1 equipped with a 40×, 1.3 NA oil objective and an Andor Ixon camera, at a frequency of 0.2 Hz. The K^+^ free recording solution (0 mM K^+^) had the same composition, except that the NaCl and KCl concentrations were 114 mM and 0 mM, respectively. After the 0 mM K^+^ recording solution was replaced with normal recording solution, recovery to basal level was monitored until a plateau was reached. At the end of each experiment, neurons were super-fused with Na^+^ calibration solutions containing stepwise increasing concentrations of Na^+^ (0, 10, 40, and 100 mM) in the presence of 3 μM gramicidin, 10 μM monensin, and 1 mM ouabain until a plateau was reached. Na^+^ calibration solutions contained [Na^+^ + K^+^] = 165 mM, 136 mM gluconate, 1.2 mM MgSO4, 0.78 mM KH2PO4, 20 mM HEPES, 1.3 mM CaCl2, pH adjusted to 7.2 with KOH. In each experiment, 3–5 ROI were selected around primary or secondary dendrites, 50–150 μm away from the soma, from 1–3 cells (using ImageJ). The fluorescence levels for each ROI were measured against time. The Na^+^ data were then analyzed using custom-written code in MATLAB (MathWorks, Natick, MA, USA). The fluorescence was corrected for bleaching, smoothed using a 5-point moving average, and transformed into calibrated Na^+^ values (in mM) using the data obtained from the calibration procedure. The Na^+^ extrusion rate was defined as the absolute value of the maximum slope of the recovery curve divided by the value of the Na^+^ peak.

### 4.10. Statistics

Statistical significance of differences for small samples or discontinuous data was tested with a non-parametric Mann–Whitney U test. Significance for two normally distributed groups was tested with an unpaired two-tailed *t*-test.
